# Atovaquone Suppresses the Growth of Metastatic Triple-Negative Breast Tumors in Lungs and Brain by Inhibiting Integrin/FAK Signaling Axis

**DOI:** 10.3390/ph14060521

**Published:** 2021-05-28

**Authors:** Nehal Gupta, Sanjay K. Srivastava

**Affiliations:** 1 Department of Biomedical Sciences, Texas Tech University Health Sciences Center, Amarillo, TX 79106, USA; nehal.gupta@ttuhsc.edu; 2 Department of Immunotherapeutics and Biotechnology, and Center for Tumor Immunology and Targeted Cancer Therapy, Texas Tech University Health Sciences Center, Abilene, TX 79601, USA

**Keywords:** atovaquone, metastasis, integrins, angiogenesis, drug repurposing, focal adhesion kinase, breast cancer, metastasis models, VEGF

## Abstract

Triple-negative breast cancer (TNBC) is considered to be the most aggressive and malignant neoplasm and is highly metastatic in nature. In the current study, we investigated the anti-metastatic potential of atovaquone, a protozoal drug prescribed for Pneumocystis pneumonia. We showed that atovaquone induced apoptosis and reduced the survival of several aggressive metastatic TNBC cell lines including metastatic patient-derived cells by reducing the expression of integrin α6, integrin β4, FAK, Src, and Vimentin. In order to study the efficacy of atovaquone in suppressing metastasized breast tumor cells in brain and lungs, we performed three in vivo experiments. We demonstrated that oral administration of 50 mg/kg of atovaquone suppressed MDA-MB-231 breast tumor growth by 90% in lungs in an intravenous metastatic tumor model. Anti-metastatic effect of atovaquone was further determined by intracardiac injection of 4T1-luc breast tumor cells into the left ventricle of mouse heart. Our results showed that atovaquone treatment suppressed the growth of metastatic tumors in lungs, liver and brain by 70%, 50% and 30% respectively. In an intracranial model, the growth of HCC1806-luc brain tumors in atovaquone treated mice was about 55% less than that of control. Taken together, our results indicate the anti-metastatic effects of atovaquone in vitro and in vivo in various breast tumor metastasis models.

## 1. Introduction

Breast cancer represents one of the most commonly occurring cancer in women worldwide and is the second leading cause of cancer-related mortality [1]. Triple-negative breast cancer (TNBC) is classified by the absence of estrogen, progesterone and human epidermal growth receptor 2 (HER2); rendering it the most lethal and resistant subtype of breast cancer [2,3]. It is considered to be a heterogeneous and aggressive disease [4,5]. TNBC accounts for 15–20% of all breast cancer patients [6]. Prior studies have validated that TNBC is more likely to metastasize to visceral organs and central nervous system than other breast cancer subtypes. Consequently, it is correlated with poor prognosis and low survival rates [7,8]. Although several advancements in diagnosis and therapeutic interventions resulted in improved survival rates, lack of effective therapeutic options for advanced and metastatic TNBC presents it as one of the greatest challenges in oncology drug development [9]. The lethality of TNBC is driven by its metastatic potential, which accounts for greater mortality rates, compared to those observed from the primary tumor. There is an urgent unmet need for the development of new treatment strategies for patients with metastatic TNBC.

Cancer metastasis is an extremely complex process and requires multiple steps, including local invasion, intravasation, and colonization to distant organs. Epithelial-mesenchymal transition (EMT) is a key driver of metastasis, which induces migratory and invasive properties by tumor cells [10]. Metastasis starts with separation of tumor cells from its primary site, which begins to invade the surrounding host tissue and finally seeded in a target organ by circulation through bloodstream or lymphatic vessels [11,12]. Studies have established that the most common metastatic sites for TNBC are lungs, liver, brain and bone [13]. Amongst these, brain metastasis is a serious threat to patient’s life because of its poor prognosis [14,15]. Moreover, treatment options are very limited for brain metastasis as most of the chemotherapeutics fail to penetrate blood-brain barrier [16]. The median survival rate with brain metastases from breast cancer is 3–5 months [17].

Integrins are transmembrane receptors that provide a link between the extracellular matrix and the cytoskeleton. They serve as adhesion molecules that facilitate cell-to-cell and cell-to-matrix interaction [18]. Importantly, two major integrins–α6 and β4 have been found to play a specific role in breast cancer progression, invasion and metastasis [19,20,21]. Upon ligand binding, integrins induce the activation of several intracellular signaling molecules including focal adhesion kinase (FAK) and steroid receptor coactivator (Src) [22,23]. The mutually activated FAK/Src complex initiates multiple downstream signaling pathways via phosphorylation of various adapter proteins that eventually lead to important cellular responses such as motility, proliferation, survival, migration and invasion [24,25]. Numerous reports have implicated the overexpression or activity of FAK/Src complex in promoting the progression of aggressive tumors, including breast tumors [26]. Henceforth, drugs targeting FAK and/or Src have been established to be effective in inhibiting tumor growth, angiogenesis and metastasis [27,28].

Anoikis is a form of programmed cell death that occurs when cells detach from the surrounding extracellular matrix (ECM). It is an essential phenomenon to maintain tissue integrity and homeostasis [29,30]. In case of metastasis, tumor cells adapt to overcome anoikis and survive detachment from the ECM. Tumor cells with metastatic potential do so by activating several survival mechanisms. Thus, tumor cells that are resistant to anoikis travel to distant sites, leading to metastasis and consequently developing secondary tumor sites [31]. Hence, suppression of anoikis resistance can have substantial implications to staunch breast cancer metastasis.

Angiogenesis is considered as one of the hallmarks of cancer [32]. It necessitates the formation of new blood vessels from the existing ones to support tumor growth. Vascular endothelial growth factor (VEGF) is a key regulator of angiogenesis [33]. Out of several VEGF ligands, VEGF-A is an important cytokine that is widely expressed by tumor cells. Interaction of VEGF-A with VEGR2 receptor leads to increased vascular permeability, cell migration, invasion and angiogenesis [34,35]. Since angiogenesis is a fundamental event in the process of tumor invasion and metastasis, anti-angiogenic drugs such as bevacizumab have been proven to inhibit cancer progression.

Atovaquone (ATQ)–an anti-malarial drug, has shown significant chemotherapeutic effects against leukemia, colon, hepatocellular and renal carcinoma [36,37,38,39]. These studies have shown the anticancer effects of atovaquone by various mechanisms that incudes inhibiting oxidative phosphorylation, DNA damage and inhibiting Signal transducer and activator of transcription 3 (STAT3). We have previously demonstrated the anti-cancer effects of ATQ in primary and paclitaxel-resistant breast tumors [40]. However, the anti-metastatic potential of this compound has not been explored. In the present study, we demonstrate the anti-metastatic effects of ATQ in metastatic TNBC. Our study establishes that ATQ reduces cell migration, invasion and growth of metastatic tumors as analyzed in various in vivo tumor models. To the best of our knowledge, this is the first study establishing the anti-metastatic potential of ATQ.

## 2. Results

### 2.1. ATQ Inhibits the Proliferation of Triple-Negative Breast Cancer Cells

**Figure 1:**
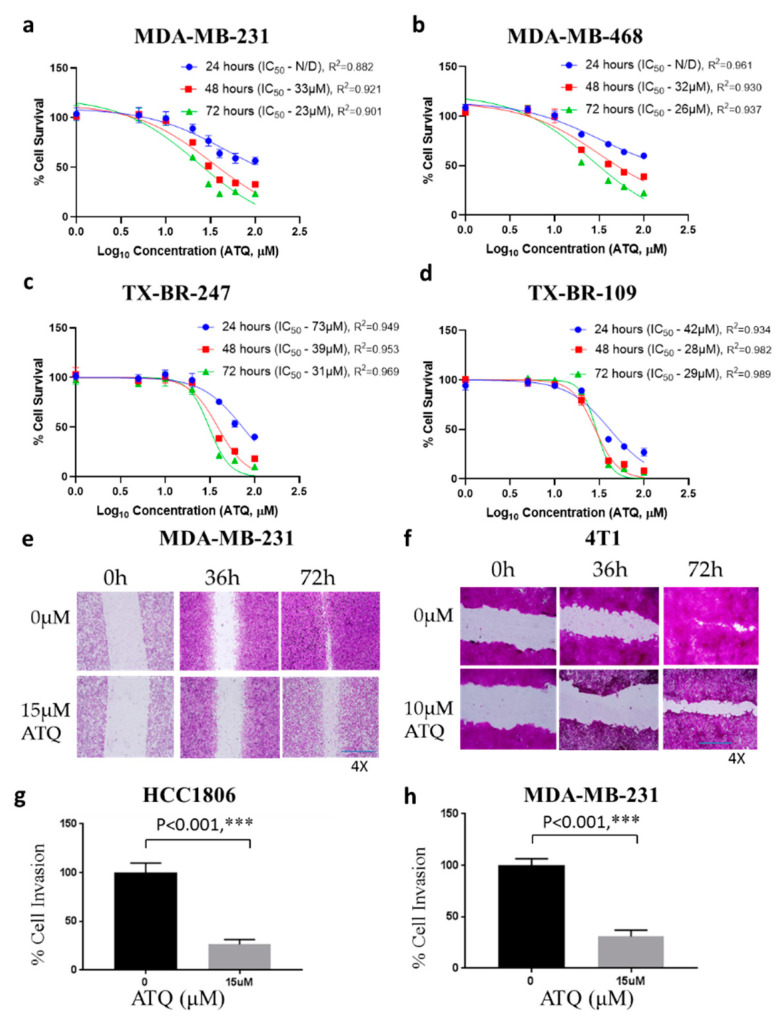
ATQ suppresses cell survival, cell migration and invasion. (**a**) MDA-MB-231, (**b**) MDA-MB-468, (**c**) TX-BR-247, and (**d**) TX-BR-109 cells were treated with varying concentrations of ATQ (0 μM, 5 μM, 10 μM, 20 μM, 30 μM, 40 μM, 60 μM, 100 μM) for 24, 48 and 72 h. Following treatment, percentage cell survival was measured by sulforhodamine B assay. The experiments were repeated three times with 4–8 replicates in each experiment. The IC50 value of ATQ was determined by fitting a sigmoidal dose–response curve to the data, using GraphPad Prism. Wound healing assay was performed with ATQ treatment at varying time points. Representative images of wounds from control and ATQ treatment in (**e**) MDA-MB-231 and (**f**) 4T1 cells. Invasion transwell assay in (**g**) HCC1806 and (**h**) MDA-MB-231 cells using Boyden’s chamber. Invading potential of the cells in the treatment and control group was analyzed by SRB assay. Values were plotted as means ± SD. *** Statistically significant as compared with control.

Considering breast tumor heterogeneity, we tested the cytotoxic effects of ATQ in four different breast cancer cell lines including two patient-derived breast metastatic cell lines. Treatment of MDA-MB-231 and MDA-MB-468 breast cancer cells with increasing concentrations of ATQ significantly reduced the survival of these cells in a concentration and time-dependent manner with an IC_50_ ranging between 23–33 µM after 48 and 72 h treatment (Figure 1a,b). Interestingly, the proliferation of metastatic patient-derived TX-BR-247 and TX-BR-109 cells was inhibited by ATQ treatment. TX-BR-247 is a triple negative cell line (estrogen, progesterone and HER2 receptor negative), whereas TX-BR-109 cell line is HER2 null. The IC_50_ was found to be in the range of 29–31 µM after 72 h treatment in TX-BR-247 and TX-BR-109 cells suggesting ATQ is effective against patient-derived cells as well. (Figure 1c,d).

### 2.2. ATQ Reduces the Migration and Invasion of Triple-Negative Breast Cancer Cells

Migration and invasion of cancer cells are the two most essential parameters that govern tumor metastasis to distant organs. To study the effect of ATQ on cell migration, we performed wound healing assay in MDA-MB-231 and 4T1 cells. Initially, we evaluated the sub-toxic concentration of ATQ in these cells lines. For instance, 15 µM ATQ at 36 and 72 h showed 80–90% cell survival in MDA-MB-231 cells. Similarly, 10 µM of ATQ showed 70–80% cell viability in 4T1 cells at 36 and 72 h. Therefore, sub-toxic concentrations of ATQ were chosen to evaluate the anti-migratory potential. Our results indicated that ATQ inhibits the migration of MDA-MB-231 cells at 72 h. To confirm the anti-migratory effects of ATQ, we performed wound healing assay in another highly aggressive TNBC cell line–4T1. Our results established that the migration of ATQ treated 4T1 cells was significantly retarded as compared to control (Figure 1e,f). Next, we evaluated the effects of ATQ on cell invasion by transwell invasion assay using Boyden’s chamber. For this purpose, 15 µM ATQ was used as it showed about 80–85% cell viability in MDA-MB-231 and HCC1806 cells. As shown in Figure 1g,h, percentage of cell invasion in ATQ treated group was approximately 70% less as compared to the controls in HCC1806 and MDA-MB-231 cells. These observations indicated that ATQ treatment inhibits cell migration and invasion of breast cancer cells suggesting an anti-metastatic potential.

### 2.3. Inhibition of Integrin/FAK/Src Signaling by ATQ

**Figure 2:**
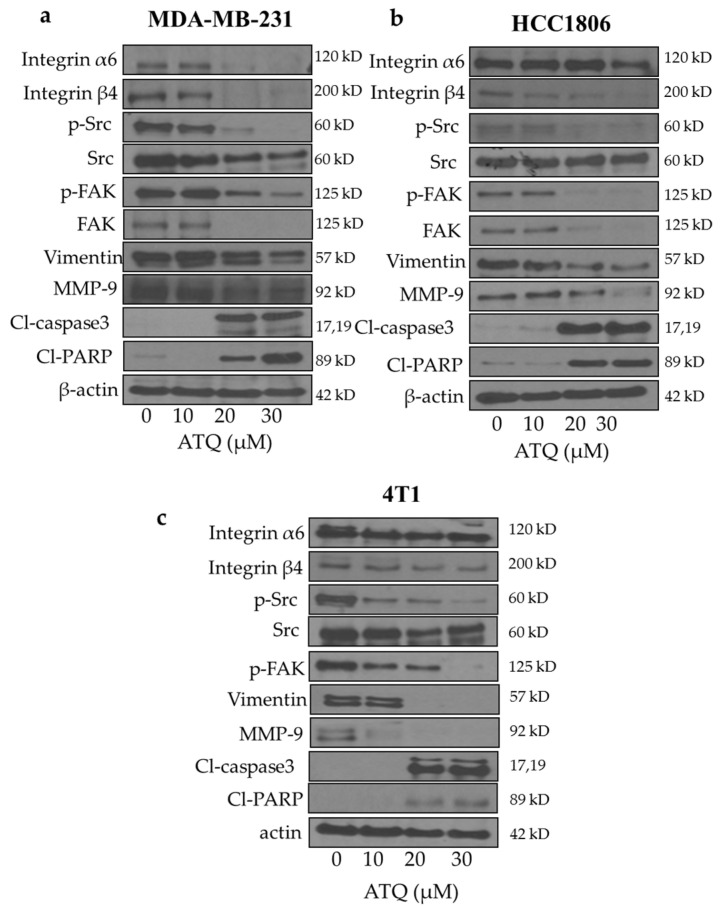
ATQ inhibits integrin-FAK signaling. (**a**) MDA-MB-231, (**b**) HCC1806, (**c**) 4T1 cells were treated with different concentrations of ATQ for 72 h. Representative blots showing concentration-dependent effect of ATQ on integrinα6, integrinβ6, p-Src, Src, p-FAK, FAK, vimentin, MMP-9, Cl caspase 3 and Cl PARP. Actin was used as loading control. Figures shown are the representative blot of at least three independent experiments.

To determine the mechanism of growth suppressive effects of ATQ, cells treated with 10, 20 and 30 µM ATQ were analyzed for the expression of integrins, FAK, Src and other metastatic markers by western blotting. The treatment of MDA-MB-231 and HCC1806 with ATQ resulted in reduced expression of integrin α6 and β4 in a concentration-dependent manner (Figure 2a,b). Surprisingly, in 4T1 murine breast cancer cells, we did not observe any significant change in integrin α6 but integrin β4 expression was significantly reduced with ATQ treatment (Figure 2c). Next, we tested the effects of ATQ on downstream signaling of integrins in breast cancer cells. Our results showed that phosphorylated FAK, phosphorylated Src and FAK levels were significantly reduced by ATQ treatment in a concentration-dependent manner in the three cell lines tested (Figure 2a,c). We also observed a notable inhibition of EMT markers–vimentin and matrix metalloproteinase 9 (MMP-9) that play a crucial role in breast cancer metastasis [41,42] with ATQ treatment. Moreover, an increase in levels of cleaved PARP and caspase-3 was observed with ATQ treatment, indicating apoptosis. These results indicate that ATQ suppresses cell motility by inhibiting integrin/FAK/Src signaling and leads to apoptotic cell death (Figure 2a–c).

### 2.4. ATQ Reduces Mammosphere Forming Efficiency of Breast Cancer Cells

**Figure 3:**
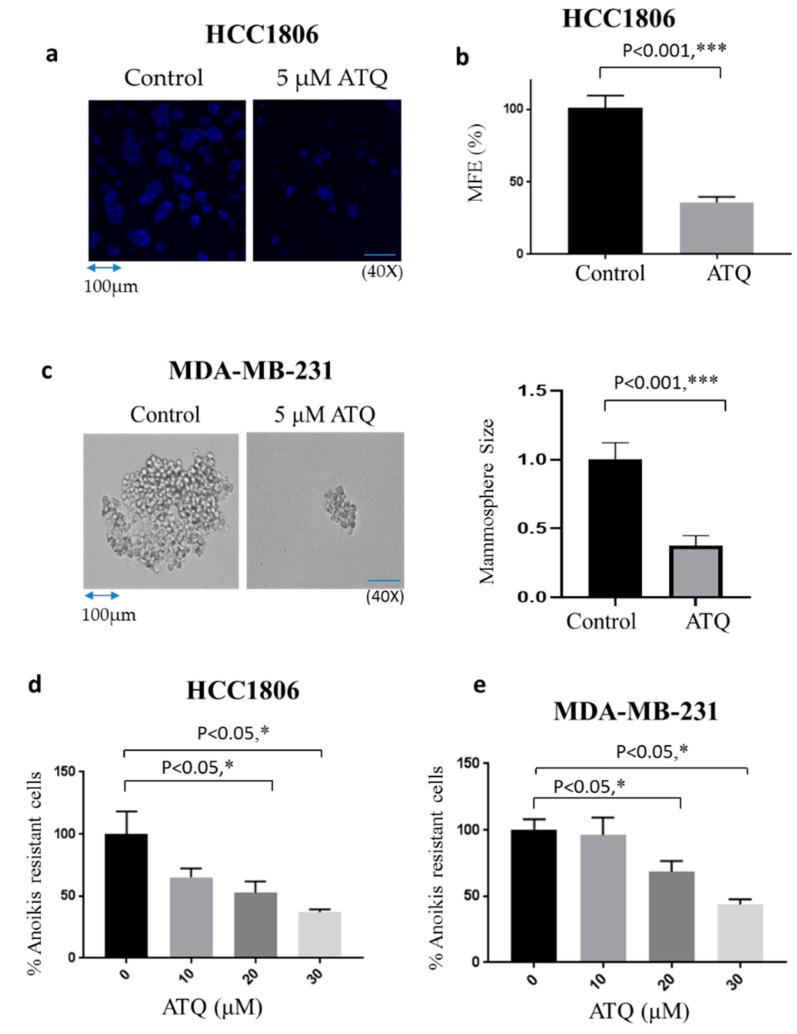
ATQ inhibits mammosphere formation and anoikis resistance in breast cancer cells. About 10,000 HCC1806 and MDA-MB-231 cells were plated in ultra-low attachment plates. Cells were pre-treated either with DMSO or 5 μM ATQ. After 10–11 days, images were taken using multiphoton confocal microscope or light microscope. (**a**) Representative image of mammosphere in control and ATQ treated HCC1806 cells and (**b**) their quantitation in terms of mammosphere forming efficiency (MFE). Blue fluorescence represents DAPI. (**c**) Representative image of mammosphere in control and ATQ treated MDA-MB-231 cells (left) and quantification of mammosphere size (right). HCC1806 and MDA-MB-231 cells were made anoikis resistant by plating in ultra-low attachment plates and further treated with varying concentrations of ATQ. Percentage inhibition in anoikis resistant cells by ATQ treatment in (**d**) HCC1806 and (**e**) MDA-MB-231 cells. Values were plotted as means ± SD. * Statistically significant as compared with control.

Mammosphere or breast tumorosphere is a three-dimensional culture system, which reflects cell heterogeneity of tumors in vitro [43]. Moreover, mammospheres are known to impart stem cell activity in breast carcinoma, have tumorigenic potential in mice and mimic in vivo tumors. Cancer stem cells including mammospheres are well known to impart drug resistance and responsible for metastasis [44]. Our results showed that treatment of HCC1806 cells with 5 μM ATQ reduced mammosphere forming efficiency (MFE) of these cells by 65%, indicating that ATQ has the potential to decrease the stemness of breast cancer cells (Figure 3a,b). To confirm these results, mammosphere assay was also performed using MDA-MB-231 cells. Consistent with other previous studies, we observed that MDA-MB-231 cells form spheroid-like structures [45,46,47], and treatment with 5 μM ATQ for 11 days suppressed the size of MDA-MB-231 spheroids (Figure 3c). These results established that ATQ was able to suppress tumor cells in the in vitro 3D culture system.

### 2.5. ATQ Reduces Anoikis Resistance of Breast Cancer Cells

Anoikis is a type of cell death, which is triggered by detachment of cells from the ECM. Various studies have shown that cancer cells need to overcome anoikis in order to acquire metastatic characteristics [48]. To determine the effect of ATQ on anoikis resistant cells, HCC1806 and MDA-MB-231 cells were made anoikis-resistant after culturing them onto ultra-low attachment plates for 48 h. These anoikis resistant cells were treated with varying concentrations of ATQ for 72 h. The treated cells were re-cultured on an adherent plate and analyzed by Sulforhodamine B (SRB) assay for cell viability. We observed that ATQ treatment group had lower cell viability as compared to dimethyl sulfoxide (DMSO)-treated control group. These results indicate that ATQ treatment reduced anoikis resistance in HCC1806 and MDA-MB-231 cells in a concentration-dependent manner (Figure 3d,e).

### 2.6. ATQ Inhibits the Growth of Metastatic Breast Tumors in Lungs

**Figure 4:**
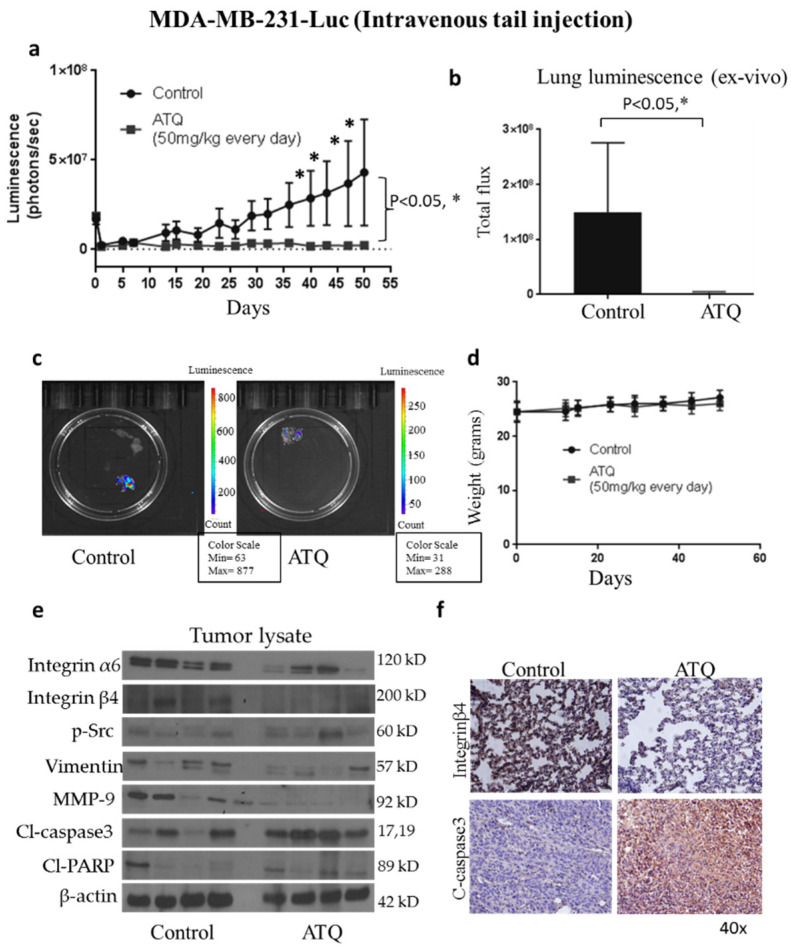
Reduction of metastatic breast tumors in lungs by ATQ through inhibition of integrin signaling in intravenous tail vein model. (**a**) Approximately, 0.5 × 10^6^ MDA-MB-231-luc cells in 1XPBS were injected intravenously in the tail vein of 4–6 week old athymic nude mice, (*n* = 8/per group). Treatment with 50 mg/kg ATQ by oral gavage everyday started at Day 7. Graph showing the luminescence in live mice from control and ATQ treated group. (**b**) Average luminescence of lungs (ex-vivo) isolated from control and ATQ treated mice at the day of termination. Values were plotted as mean ± SEM. (**c**) Representative image of lung from control and ATQ treated mice. The value of luminescence in the control and treated group ranged from 63–877 and 31–288 photons/sec respectively. (**d**) Average weight of mice from control and ATQ treated group throughout the experiment. Tumors were removed after terminating the experiment, homogenized, lysed, and analyzed for integrinα6, integrinβ6, p-Src, vimentin, MMP-9, Cl caspase 3 and Cl PARP by Western blotting. Actin was used as loading control. (**e**) Each lane of blot represents tumor from individual mouse. (**f**) Immunohistochemical staining for integrinβ6 and Cl caspase 3 in tumor lesions in lungs of control and ATQ treated mice. * Statistically significant as compared with control.

To evaluate the anti-metastatic effects of ATQ, we performed three different in vivo tumor models using three different cell lines. In the first experiment, we evaluated the efficacy of ATQ in suppressing the growth of metastasized breast tumors in lungs. Human breast cancer MDA-MB-231 cells were injected into the tail vein of athymic nude mice (*n* = 16) with the objective that most of the cells can lodge into lungs. Once tumor localization was confirmed by luminescence, mice were randomly divided into two groups with eight mice in each group. Treatment group received 50 mg/kg ATQ everyday by oral gavage. The tumor growth was monitored by measuring tumor luminescence periodically using IVIS imaging system. Representative IVIS images of mice from control and ATQ treated group at Day 50 are shown in Figure S2A. Our results showed that oral administration of ATQ significantly reduced the growth of breast tumors in lungs by 90% (Figure 4a). At the end of experiment, lungs were isolated and luminescence was recorded. We observed that the average luminescence in the lungs of ATQ treated mice was reduced by 80% as compared to the control group (Figure 4b,c). The mice were periodically weighed during the experiment. We did not observe any significant difference in the bodyweight of control vs. treated mice–indicating no obvious toxicity by ATQ treatment (Figure 4d). Tumors collected from control and treated mice were subjected to Western blot analysis. In Figure 4e, each band represents lysate from a separate tumor coming from a separate mouse. Intriguingly, remarkable suppression of Integrin α6, integrin ’β4, metastatic markers—vimentin and MMP-9, and enhanced cleavage of caspase-3 was observed in ATQ-treated tumor lysate (Figure 4e). These observations were also confirmed by IHC staining of tumors from control and ATQ-treated mice for integrin β4 and cleaved caspase-3 (Figure 4f). These results indicate that breast tumor growth suppression by ATQ in lungs was associated with inhibition of integrin signaling and induction of apoptosis.

### 2.7. ATQ Suppresses the Growth of Metastasized Breast Tumors

Breast cancer metastasizes to brain, lungs, liver and bone. After confirming that ATQ was able to suppress lung metastasized breast tumors, we wanted to evaluate the efficacy of ATQ at other metastatic sites. Before performing any in vivo experiment, we first evaluated the concentration of ATQ in plasma, tumor and brain of mice bearing HCC1806 tumors in mammary fat pad and treated with 50 mg/kg ATQ daily for 42 days to observe whether it reaches the desired site of action. Our results showed that 19.8 µg/mL ATQ in plasma, 833 ng/mL in brain and 3.4 µg/mL in tumor was achieved (Table S1A–C). These results indicate that the therapeutic concentration of ATQ can be reached by oral administration as the IC50 of ATQ ranges between 23–33 µM in multiple breast cancer cell lines (Figure 1).

**Figure 5:**
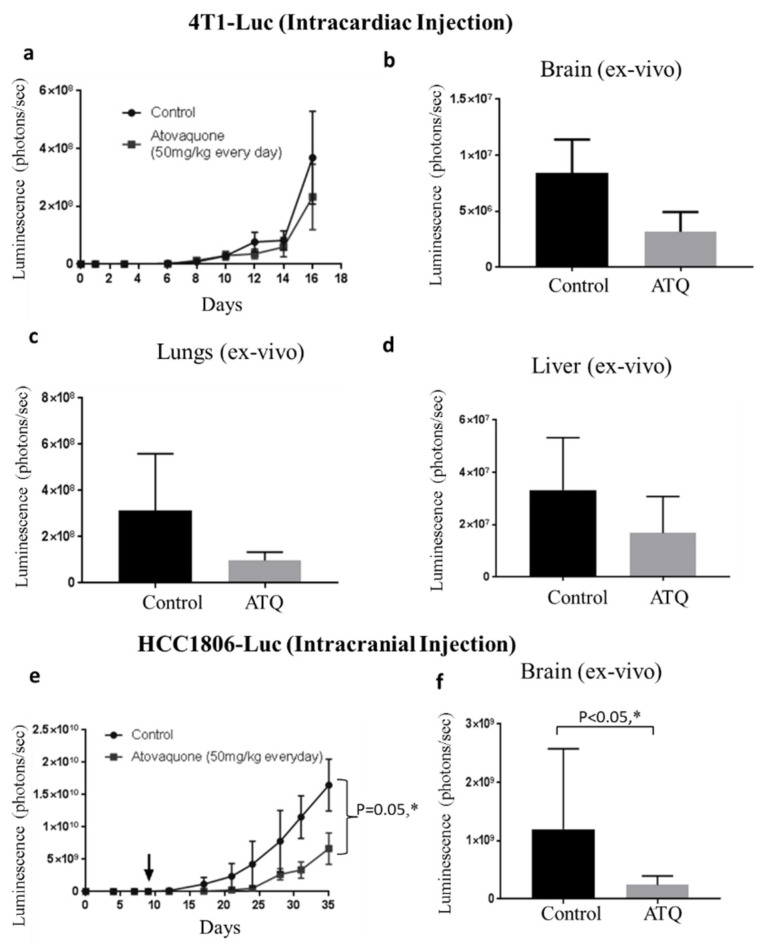
ATQ suppressed the growth of metastatic breast tumors in brain, lungs and liver in intracardiac and intracranial model. Luciferase expressing 4T1-BR cells were injected by intracardiac route in female Balb/c mice. ATQ treatment was started 24 h after cell injection and the mice were imaged periodically, (*n* = 8/per group). (**a**) Brain luminescence at different days from control and ATQ treated in live mice. (**b**) At the day of termination, mice brain from control and ATQ treated group was isolated, imaged (ex-vivo) and the average brain luminescence is plotted. (**c**) and (**d**) the average luminescence quantitated from lungs and liver (ex-vivo) respectively, from control and ATQ treated mice. Values were plotted as mean ± SEM. Intracranial model—(**e**) approximately 0.01 × 10^6^ HCC1806-luc cells were injected in the brain of 4–6 weeks old athymic nude mice. Treatment with 50 mg/kg ATQ by oral gavage everyday started 7 days post tumor cells injection, till Day 35. Values were plotted as means ± SEM. (*n* = 9/group). (**f**) Luminescence of isolated brain from control and ATQ-treated mice at Day 35. Values were plotted as mean ± SEM. * Statistically significant as compared with control.

Following this, we performed an experiment where luciferase expressing 4T1-BR cells were injected by intracardiac route into the left ventricle of mouse heart. The 4T-1 cells are highly aggressive and considered to be indicative of late stage breast cancer [49]. After confirming the luminescence in brain, treatment with 50 mg/kg ATQ was started after 24 h of intracardiac injection in the treatment group. The treatment continued for 16 days by oral gavage daily. Due to the metastatic breast tumor burden in brain, experiment was terminated at Day 16. Our results indicated that luminescence in the brain was reduced by about 30% by the end of experiment in ATQ treated group (Figure 5a). The insignificant difference in luminescence of control and treated mice could be due to large variations in the luminescence of tissues from control group, and could also be due to the short duration of treatment as compared to the other experiments we performed. At the end of the experiment, brain, lungs and liver were dissected out and imaged for luminescence. We observed about 35% reduced signal in the isolated brains of ATQ treated group as compared to control group (Figure 5b). The lungs and livers were also imaged for luminescence from both the groups and the treatment group showed a reduction of 60% and 30% in tumor growth in lungs and liver respectively (Figure 5c–d). Although metastatic tumor growth reduction in brain and liver was not statistically significant, we see a clear decrease in the luminescence in ATQ treated group indicating inhibitory effects of ATQ in metastasized tumors. These results suggest that ATQ suppresses the growth of metastasized breast tumors in brain, lungs and liver.

### 2.8. ATQ Inhibits the Growth of HCC1806 Tumors in Intracranial Tumor Model

The in vivo efficacy of ATQ was further validated in an intracranial model. In this experiment, human breast cancer HCC1806-luc cells were injected in the brain of 18 athymic nude mice. Once each mouse attained tumor, mice were segregated into two groups with 9 mice in each group. ATQ (50 mg/kg) was given orally every day to the mice in treatment group and the growth of the tumors was monitored. Our results showed that the growth of tumors in ATQ-treated group was reduced significantly as compared to control group. At Day 35, luminescence in the treated group was reduced by 55% as compared to control group (Figure 5e). Figure S2B shows representative IVIS images of mice from control and ATQ treated group at Day 35. We also analyzed luminescence in the isolated brains from both groups after euthanizing the mice. Our results showed a clear suppression of tumor growth by ATQ treatment, as indicated by the reduced luminescence in ATQ treated brains as compared to controls (Figure 5f).

### 2.9. ATQ Inhibits In Vivo Breast Tumor Angiogenesis

**Figure 6:**
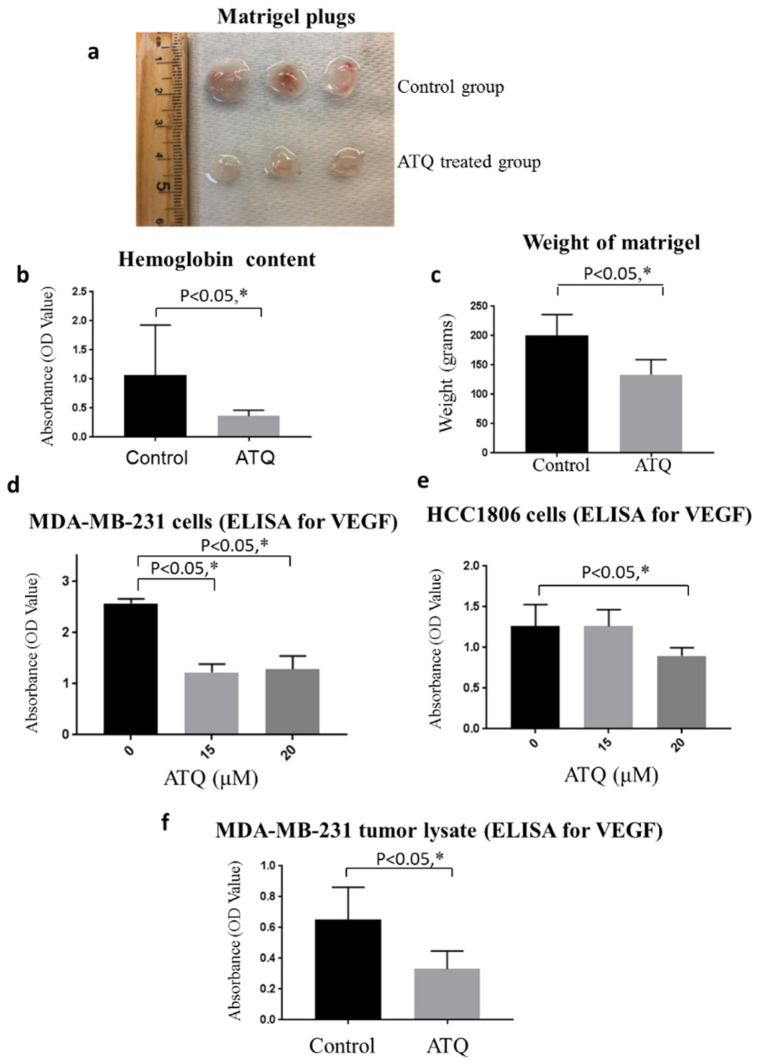
Inhibition of angiogenesis and VEGF levels by ATQ. Matrigel plug-bearing mice were fed with 50 mg/kg ATQ daily for 12 days. (**a**) Images of matrigels isolated from control and ATQ treated groups at Day 12. (**b**) Plugs were collected and analyzed for hemoglobin content by Drabkin’s reagent. (**c**) Average weight of matrigel plugs from control and ATQ treated mice. VEGF secretion was estimated by performing ELISA assay in (**d**) MDA-MB-231 and (**e**) HCC1806 cell supernatant treated with either DMSO control or varying concentrations of ATQ. (**f**) ELISA assay for VEGF in the tumor lysates obtained from control and ATQ treated mice group. Values were plotted as means ± SD. * Statistically significant as compared with control.

Over 45 years, the role of angiogenesis in tumor growth and metastasis has been studied and validated as an essential component of tumor metastasis [50]. To understand whether ATQ directly affects tumor angiogenesis, we performed in vivo matrigel plug assay in female athymic nude mice. The dissected plugs showed that ATQ treatment decreased angiogenesis macroscopically, due to decreased redness of the ATQ-treated plugs (Figure 6a). Our results showed a 65% reduction in hemoglobin content in ATQ treated plugs as compared to untreated plugs (Figure 6b). Interestingly, we found that the average weight of plugs in treated group was significantly less when compared to control group; suggesting inhibition of angiogenesis by ATQ (Figure 6c).

### 2.10. ATQ Inhibits Secretion of VEGF from Breast Tumor Cells

The role of VEGF in migration, invasion and neovascularization is well established in tumor microenvironment [33]. Since we observed inhibition of angiogenesis by ATQ, therefore, we next examined whether ATQ suppresses the secretion of VEGF in cell supernatant and its synthesis in tumor cells by ELISA assay. ATQ treatment significantly inhibited secreted levels of VEGF in MDA-MB-231 and HCC1806 breast cancer cells in a dose-dependent manner. Treatment with 30 µM ATQ inhibited VEGF secretion in MDA-MB-231 and HCC1806 cells by 50% and 30% respectively (Figure 6d,e). Similarly, VEGF production was also evaluated in the tumor lysates obtained from control and ATQ treated mice. We observed that ATQ treated tumors had significantly less VEGF levels as compared to control tumors (Figure 6f). These results indicate angiogenesis suppression by ATQ by inhibiting VEGF production and secretion.

## 3. Discussion

TNBC is one of the leading causes of breast cancer-related deaths worldwide. It is mostly considered untreatable due to the absence of targets and is highly metastatic in nature. Unlike other breast cancer subtypes, there is no effective targeted therapy for TNBC, rendering it very difficult to treat by conventional therapeutic options [51]. Therefore, an alternative treatment approach is much needed to control this malignancy.

The studies presented herein rigorously evaluated the anti-metastatic potential of an anti-malarial drug ATQ in metastatic TNBC, a breast cancer subtype that is currently untreatable. We examined the effects of ATQ in multiple breast cancer cell lines and in a variety of mouse tumor metastatic models. We demonstrated that ATQ treatment reduced the proliferation of TNBC cells including patient-derived metastatic breast cancer cells in a time and concentration-dependent manner. Our results also showed suppression of cell migration and invasion of breast cancer cells by ATQ treatment, both of which are considered crucial steps in metastasis.

Integrins are the master regulators of various cellular processes. There is a universal agreement that integrin α6 and β4 play a pivotal role in breast cancer progression [52]. Lu et al. have shown that the expression of integrin β4 is restricted and is associated with basal-like (TNBC) cancers [53]. Our studies established that ATQ treatment induced caspase-mediated apoptosis by inhibiting integrin α6β4 signaling axis in breast cancer cells. Integrin signaling is known to be mediated by downstream activation of FAK and Src. Catalytic activities of FAK and Src are crucial in tumor metastasis and angiogenesis of several neoplasms including breast cancer, making them potential targets for therapy [54,55]. Interestingly, ATQ treatment inhibited the expression as well as activation of these downstream proteins in all the breast cancer cell lines tested. PI3K-Akt or RAS-MEK-ERK or STAT3-c-Myc pathways; downstream to integrin-FAK-Src signaling were not evaluated in the current study and would be the topic of further investigation. MMPs are capable of degrading numerous matrix components. Amongst many MMPs, MMP-9 plays a major role in degradation of ECM. Vimentin is considered as a characteristic marker for cells undergoing epithelial-mesenchymal transition. Therefore, both of the aforementioned proteins play a pivotal role in angiogenesis and tumor metastasis [56,57,58]. Our results revealed that ATQ reduced the constitutive expression of vimentin and MMP-9 suggesting ATQ inhibits metastatic markers in breast cancer.

Cancer cells metastasize only after acquiring resistance to anoikis, thus, inhibiting the growth of anoikis-resistant cells could help prevent metastasis of malignant cells [59]. Interestingly, studies from the groups of Martin Schwartz and Steve Frisch showed that integrin-dependent signaling is required to suppress anoikis [60]. We observed that ATQ treatment reduced anoikis resistance in MDA-MB-231 and HCC1806 cell lines; an observation that can be attributed to inhibition of integrin signaling by ATQ. Mammospheres are 3-D culture models that mimic tumor growth in vitro [61]. We observed that ATQ treatment reduced mammosphere forming efficiency of HCC1806 and MDA-MB-231–TNBC cell lines. Furthermore, we evaluated the effects of ATQ on tumor angiogenesis. Our results revealed that new blood vessel formation on matrigel was significantly inhibited by ATQ treatment, providing a critical clue to the ability of ATQ to inhibit angiogenesis. However, more studies are needed to further substantiate this observation.

To determine the anti-metastatic efficacy of ATQ in vivo, we used three different tumor models using three different TNBC cell lines. Our results clearly demonstrated strong anti-metastatic effects of ATQ in lungs, brain and liver, the primary metastatic sites for breast tumor. It is noteworthy that ATQ treatment suppressed metastasized MDA-MB-231 breast tumors in lungs by 90%. Suppression of metastatic breast tumors in lungs by ATQ was associated with inhibition of integrin signaling as demonstrated by Western blot and immunohistochemical staining of tumors, and consistent with our in vitro observations. Furthermore, we used a highly aggressive cell line 4T1–representing Stage IV breast cancer, which is considered to be untreatable and performed intracardiac injection using this cell line. Injection of 4T1-BR cells by intracardiac route resulted in metastases of these cells in lungs, brain and liver; representing it as an appropriate model for breast cancer metastasis. Our results showed that ATQ treatment with 50 mg/kg every day inhibited the growth of metastasized tumors in the brain, lungs and liver. However, the inhibitory effect of ATQ in intracardiac model was not as vigorous as compared to i.v. tail vein metastasis model. The reason behind such discrepancy in the efficacy of ATQ in different models is not clearly understood at this point. One possible reason could be the short duration of treatment in case of intracardiac model. Studies revealed that brain metastases develop among 20% of all cancer patients with a higher incidence in breast, colorectal and lung carcinoma. Therefore, we evaluated the efficacy of ATQ in suppressing metastatic breast tumor growth in brain. Intriguingly, ATQ suppressed the growth of HCC1806 breast tumors in brain by 55% as evaluated in an intracranial model.

Prior studies have shown the anti-cancer effects of ATQ by inhibiting STAT3 and mitochondrial respiration [36,37,39]. In our previous studies, we observed that ATQ significantly suppressed the primary and paclitaxel resistant breast tumor growth by inhibiting HER2/β-catenin/c-Myc signaling, supporting our rationale for the current investigation [40]. We, among others, have shown that HER2 regulates integrins in different cancer models [62,63]. Thus, in line with previous studies, our current study showed ATQ suppresses metastasized breast tumor growth by inhibiting integrin-FAK-Src signaling with reduction in the markers of metastasis. Herein, we established the first in vitro and in vivo evidence for the anti-metastatic potential of an anti-malarial drug–ATQ in metastatic TNBC.

ATQ is available in clinic with doses ranging from 100–750 mg/day based on the type of disease. Currently, ATQ is in clinical trials for lung carcinoma and acute myeloid leukemia (NCT02628080, NCT03568994). It is important to note that in our study, 50 mg/kg ATQ after 60 days of treatment did not show any signs of toxicity as analyzed by comprehensive blood chemistry analysis from control and ATQ treated mice (Figure S1). Moreover, the translational consequences of these findings are substantial. It is important to note that human equivalent dose of ATQ used in our study is 250 mg which is about four fold less than its clinically recommended dose. This report clearly establishes the anti-metastatic potential of ATQ, an effect that has not been a subject of investigation in prior studies. In conclusion, our study provides convincing results to establish the strong anti-tumor and anti-metastatic effects of ATQ in TNBC. Taken together, findings from the current study have the potential to lay a foundation for repurposing ATQ for TNBC treatment.

## 4. Materials and Methods

### 4.1. Cell Culture and Reagents

Human triple-negative breast carcinoma cell lines MDA-MB-231, HCC1806 and MDA-MB-468 and murine 4T1 cells were cultured in DMEM supplemented with 10% FBS (fetal bovine serum) and 5% PSN (penicillin–streptomycin–neomycin). MDA-MB-231 and 4T-1-luc cells were purchased from ATCC and PerkinElmer (Waltham, MA, USA) respectively. HCC1806 cells were a kind gift from Dr. Sophia Ran, Southern Illinois University-School of Medicine. MDA-MB-468 cells were obtained from MD Anderson, Houston, Texas. The HCC1806-luc cell line was generated in our lab using lentiviral transfection reagents and puromycin as a selection reagent. All cell lines were authenticated by STR analysis by our core facility before performing any experiment. Patient derived cells (TX-BR-247 and TX-BR-109) were obtained from Children’s Oncology Group (Texas Tech University Health Sciences Center, Lubbock, TX, USA). These cells were maintained in IMDM supplemented with 20% FBS, 1% PSN and 1X ITS (5 μg/mL insulin, 5 μg/mL transferrin, 5μg/mL selenous acid). For in-vitro studies, atovaquone (#A7986) was purchased from Sigma-Aldrich (St. Louis, MO, USA). DMSO was used as solvent to dissolve the powdered atovaquone. The final concentration of DMSO in cell culture was in the range of 0.01–0.5%. Equal amount of DMSO was added in the respective control group to nullify any DMSO-specific effects. Atovaquone suspension (Mepron) was purchased from Prasco laboratories for in vivo studies.

### 4.2. Sulphorhodamine Assay

Cells were plated at a density of 4000–5000/well and left overnight for attachment. The next day, cells were treated with varying concentrations of ATQ for 24, 48 and 72 h. At the desired time point, cells were fixed with 10% trichloroacetic acid (TCA) and left at 4 °C overnight. The cells were then stained with SRB dye for 1–2 h after washing with distilled water. Further, plates were washed with 1% solution of acetic acid and the optical density was measured in 10 mM Tris-base solution at 570 nm, using plate reader (BioTek Instruments, Winooski, VT, USA) [64]. The IC50 value of ATQ was determined by performing a non-linear regression analysis and fitting a sigmoidal dose–response curve to the data, using the GraphPad Prism.

### 4.3. Wound Healing

MDA-MB-231 and 4T1 cells were plated at a density of 0.3 × 10^6^ cells/well and incubated to form a monolayer in 6-well dishes. Once a uniform monolayer was formed, wound was created by scratching the monolayer with a 1ml sterile tip. Floating cells were removed by washing the cells with PBS three times. Further, media was added in all the wells with drug addition in the treatment group. The cells were fixed using 10% TCA at desired time points and stained with 0.4% (*w*/*v*) SRB dye. The wound was imaged using bright field microscope (Olympus Inc, Center Valley, PA, USA).

### 4.4. Cell Invasion Assay

Boyden’s chamber (BD Biosciences, San Jose, CA, USA) was used to perform cell invasion assay. Wells of Boyden’s chamber was coated with matrigel. Briefly, cells were starved in serum-free media for about 24 h. The next day, serum starved cells were seeded in the upper well of Boyden’s chamber and the lower chamber was filled with complete media containing 10% FBS and VEGF as chemo-attractants. After 4 h of cell seeding, 15 µM ATQ was added to upper chamber of the well, whereas control wells were treated with vehicle (DMSO). After 72h of ATQ treatment, cells from the upper side of membrane were removed whereas cells that migrated to the lower side of the membrane were fixed with 10% TCA and stained with 0.4% (*w*/*v*) SRB solution. The SRB dye was solubilized in 10 mM Tris buffer and the absorbance was taken using a microplate reader (BioTek Instruments, Winooski, VT, USA) as described previously in [65].

### 4.5. Western Blotting

MDA-MB-231, HCC1806 and 4T1 cells were exposed to varying concentrations of ATQ for 72 h. Cells were collected, lysed, and approximately 30–50 μg of protein was subjected to SDS-gel electrophoresis followed by immunoblotting as previously described by us [66,67]. The antibodies integrinα6 (#3750, rabbit mAb), p-Src (#6943, rabbit mAb), Src (#2108, rabbit mAb), p-FAK (#3283, rabbit mAb), Vimentin (#3932, rabbit mAb), MMP9 (#2270, rabbit mAb), Cl-caspase3 (#9664, rabbit mAb), Cl-PARP (#9541, rabbit mAb) were purchased from Cell Signaling Technologies (Danvers, MA, USA). Integrinβ4 (#sc377523, mouse mAb), FAK (#sc1688, mouse mAb) was purchased from Santa Cruz Biotechnology and β-actin (#A5441, mouse mAb) was purchased from Sigma (St. Louis, MO, USA).

### 4.6. Mammospheres Assay

Approximately 10,000 HCC1806 and MDA-MB-231 cells were cultured in mammospheres media at 37 °C and 5% CO_2_ for 10–11 days with or without ATQ. Mammospheres medium contains serum free DMEM/F12 supplemented with 10 ng/mL FGF (fibroblast growth factor), 20 ng/mL EGF (epidermal growth factor), 1xITS (insulin–transferrin–selenium) and a B27 supplement. For HCC1806 cells, at Day 10, images were taken using confocal microscope in control and treatment group. Mammospheres forming efficiency (MFE%) was calculated using the following equation: MFE (%) = (Number of mammospheres per well)/(Number of cells seeded per well) x 100 [68]. For MDA-MB-231 cells, at Day 11, images were taken using light microscope in control and treatment group. Further, the size of the mammosphere (diameter) was calculated using image J software.

### 4.7. Anoikis Assay

Anoikis assay was performed as described by us previously with few modifications [69]. Briefly, 1 × 10^6^ cells were plated in ultra-low attachment plates. After 48 h, cells were counted using trypan blue and were re-plated again in ultra-low attachment plates. These cells were considered as anoikis resistant cells. Treated wells were exposed to various concentrations of ATQ whereas same concentration of DMSO was added to control wells. After 72 h, cells were centrifuged at 1000 rpm for 5 min and uniformly distributed in a 24-well plate and incubated at 37 °C in an incubator. Once the cells were attached (6–8 h), plates were processed for SRB assay and absorbance value was noted. Reduction in survival is represented as a percentage decrease in anoikis resistance.

### 4.8. Tumor Therapy Model

All animal experiments were carried out according to approved Institutional Animal Care and Use Committee (IACUC) (protocol #19181) at the Texas Tech University Health Sciences Center (Amarillo, TX, USA). Mice were allowed to acclimatize in the animal facility for at least 7 days before use and maintained under specific pathogen-free conditions. The anti-malarial dose of atovaquone used clinically is 1000 mg (16.6 mg/kg), which when converted to mice dose comes out to be 200 mg/kg. We further reduced this dose four times and the final dose of atovaquone used for the current study was 50 mg/kg. Living image software version 4.7 (Perkin Elmer, Waltham, MA, USA) was used to analyze the images obtained from IVIS imaging system

### 4.9. In Vivo Metastasis Model (Intravenous Tail Vein Injection)

MDA-MB-231 cells with luciferase expression were collected and washed with PBS. A 100 μL cell suspension containing 0.5 × 10^6^ cells were injected in the tail vein of 16 immunodeficient female athymic nude mice (4–6 weeks old). Each mouse was imaged using IVIS imaging system (Perkin Elmer, Waltham, Massachusetts) to get the base value after intra-peritoneal injection of 3 mg luciferin (Goldbio. St. Loius, MO, USA) in 100 µL of sterile water. Mice were divided into two groups with 8 mice in each group. After 7 days of injection, the treatment group received 50 mg/kg ATQ by oral gavage daily, whereas control group received vehicle only. The mice were humanely sacrificed at Day 60 as the control mice started showing signs of sickness. The lungs from control and treated mice group were collected, imaged for luminescence and kept for further analysis.

### 4.10. Metastasis Model by Intracardiac Injection

Female Balb/c mice (4–6 weeks old) were obtained from Charles River (Wilmington, MA, USA) and maintained as per the IACUC guidelines. For the metastatic breast cancer model, we used 4T1-BR (brain seeking) cells and injected these cells in the heart of each mouse. This method was first described by Conley and later by other groups including our lab [70,71]. In this model, exponentially growing 4T1-BR luciferase cells (2.5 × 10^4^ cells/50 µL) were harvested, washed and injected into the heart’s left ventricle of 20 mice using stereotaxic apparatus. The luminescence from cells injected was observed in brain within 5 min of intracardiac injection of cells. After confirming the luminescence in 16 mice out of 20, mice were randomly divided into two groups with 8 mice in each group. The mice without luminescence were excluded from the study. Treatment group received 50 mg/kg ATQ everyday by oral gavage. The luminescence signal from mice was used to analyze rate and extent of metastasis. The experiment was terminated at Day 16 by humanely euthanizing the mice with CO_2_ overdose followed by cervical dislocation. Mice brain, lungs and liver were carefully dissected, weighed and imaged for luminescence.

### 4.11. Intracranial Tumor Model

Intracranial injection was performed as previously described by us [65]. HCC1806-luciferase expressing cells were used for this purpose and these cells were injected in 4–6 weeks old female athymic nude mice. Approximately 0.1 × 10^6^ cells in a cell suspension of 5 µL were injected in the brain of each mouse using stereotaxic apparatus at a flow rate of 1 µL/min. After dividing the mice into two groups (*n* = 9), 50 mg/kg ATQ was administered orally every day in treated group. The tumor growth in mice brain was monitored through a non-invasive imaging technique. At Day 35, mice were humanely sacrificed by CO_2_ overdose, and brains from control and treated group was dissected out and imaged for luminescence using IVIS imaging.

### 4.12. Matrigel Plug Assay

Matrigel plug assay was performed as described earlier [72]. Approximately 250 µL matrigel (BD Biosciences, Bedford, MA, USA) containing 40 ng/mL VEGF and 10 ng/mL FGF were injected subcutaneously into 6–8-week-old female athymic nude mice. The injected matrigel rapidly formed a single, solid gel plug. Starting from next day, mice were gavaged with 50 mg/kg ATQ daily for 12 days, after which they were euthanized and the matrigel plugs were recovered. The plugs were dispersed in PBS. Hemoglobin content in matrigel was measured using Drabkin’s reagent (Sigma-Aldrich, St. Louis, MO, USA) according to manufacturer’s instructions, to quantify blood vessel formation.

### 4.13. Estimation of VEGF Secretion by ELISA

ELISA assay was performed for VEGF using ELISA kit (Invitrogen Corp., Carlsbad, CA, USA) according to manufacturer’s instructions. Secreted VEGF levels in control cells and ATQ treated cells (72 h) was estimated in cell culture supernatant. For the VEGF level in tumor lysate, MDA-MB-231 tumors were collected from control and atovaquone treated mice at the end of the experiment. Tumors were lysed using RIPA buffer and protein was estimated using Bradford reagent (Bio-Rad). Equal amounts of protein (60–70 μg) from control and atovaquone treated tumor lysate samples were used to perform ELISA assay.

### 4.14. Statistical Analysis

The statistical calculations and analysis were performed using Prism 7.0 (GraphPad software Inc., San Diego, CA, USA). Results represent means ± SD or S.E.M. of at least three independent experiments. Data was analyzed by Student’s *t*-test or Fischer (F) test. Differences were considered statistically significant at *p* < 0.05.

## Data Availability

All the data generated during this study are included in this article or supplementary material.

## Supplementary Materials

The following are available online at https://www.mdpi.com/article/10.3390/ph14060521/s1, Figure S1: ATQ treatment does not cause any major side effect in chronic toxicity model. The 50 mg/kg ATQ was administered by oral route every day to mice. After 60 days, mice were sacrificed; plasma was collected and sent to Texas Veterinary Medical Diagnostic Laboratory System, Amarillo, TX for analysis. (A) Total serum, (B) albumin, (C) calcium, (D) BUN, (E) phosphorus, and (F) glucose. Values were plotted as means ± SD. Figure S2: representative IVIS images from intravenous and intracranial model. (A) Representative IVIS images of mice from Intravenous tail injection using MDA-MB-231 luc cells. Images were taken at Day 50 from the control and ATQ treated groups. The value of luminescence in the control group ranged from 230–4354 photons/sec, whereas in the treated group, it was from 31–324 photons/sec. (B) Representative IVIS images of mice from HCC1806 intracranial injection at Day 35. Luminescence value in the control and treated group ranges from 2480–46,605 and 667–11,746 photons/sec, respectively. Table S1A–C: quantitation of ATQ concentration by LC-MS. (A) Samples indicating concentration of ATQ in (A) plasma, (B) brain and (C) tumor.

## References

[1] Sharma G.N., Dave R., Sanadya J., Sharma P., Sharma K. (2010). Various types and management of breast cancer: An overview. J. Adv. Pharm. Technol. Res..

[2] Al-Mahmood S., Sapiezynski J., Garbuzenko O.B., Minko T. (2018). Metastatic and triple-negative breast cancer: Challenges and treatment options. Drug Deliv. Transl. Res..

[3] Herold C.I., Anders C.K. (2013). New targets for triple-negative breast cancer. Oncology.

[4] Chiu A.M., Mitra M., Boymoushakian L., Coller H.A. (2018). Integrative analysis of the inter-tumoral heterogeneity of triple-negative breast cancer. Sci. Rep..

[5] Pareja F., Geyer F.C., Marchiò C., Burke K.A., Weigelt B., Reis-Filho J.S. (2016). Triple-negative breast cancer: The importance of molecular and histologic subtyping, and recognition of low-grade variants. NPJ Breast Cancer.

[6] Blows F.M., Driver K.E., Schmidt M.K., Broeks A., Van Leeuwen F.E., Wesseling J., Cheang M.C., Gelmon K., Nielsen T.O., Blomqvist C. (2010). Subtyping of breast cancer by immunohistochemistry to investigate a relationship between subtype and short and long term survival: A collaborative analysis of data for 10,159 cases from 12 studies. PLoS Med..

[7] Dent R., Hanna W.M., Trudeau M., Rawlinson E., Sun P., Narod S.A. (2009). Pattern of metastatic spread in triple-negative breast cancer. Breast Cancer Res. Treat..

[8] Tseng L., Hsu N., Chen S., Lu Y., Lin C., Chang D., Li H., Lin Y., Chang H., Chao T. (2013). Distant metastasis in triple-negative breast cancer. Neoplasma.

[9] Kozlowski J., Kozlowska A., Kocki J. (2015). Breast cancer metastasis-insight into selected molecular mechanisms of the phenomenon. Postepy Hig. I Med. Dosw..

[10] Wei S.C., Fattet L., Yang J. (2015). The forces behind EMT and tumor metastasis. Cell Cycle.

[11] Hunter K.W., Crawford N.P., Alsarraj J. (2008). Mechanisms of metastasis. Breast Cancer Res..

[12] Talmadge J.E., Fidler I.J. (2010). AACR centennial series: The biology of cancer metastasis: Historical perspective. Cancer Res..

[13] Weigelt B., Peterse J.L., Van’t Veer L.J. (2005). Breast cancer metastasis: Markers and models. Nat. Rev. Cancer.

[14] Fokstuen T., Wilking N., Rutqvist L.E., Wolke J., Liedberg A., Signomklao T., Fernberg J.-O. (2000). Radiation therapy in the management of brain metastases from breast cancer. Breast Cancer Res. Treat..

[15] Evans A., James J., Cornford E., Chan S., Burrell H., Pinder S., Gutteridge E., Robertson J., Hornbuckle J., Cheung K. (2004). Brain metastases from breast cancer: Identification of a high-risk group. Clin. Oncol..

[16] Gounder M.M., Spriggs D.R. (2011). Inclusion of patients with brain metastases in phase I trials: An unmet need. Clin. Cancer Res..

[17] Boogerd W., Vos V., Hart A., Baris G. (1993). Brain metastases in breast cancer; natural history, prognostic factors and outcome. J. Neuro Oncol..

[18] Clark E.A., Brugge J.S. (1995). Integrins and signal transduction pathways: The road taken. Science.

[19] Diaz L.K., Cristofanilli M., Zhou X., Welch K.L., Smith T.L., Yang Y., Sneige N., Sahin A.A., Gilcrease M.Z. (2005). β4 integrin subunit gene expression correlates with tumor size and nuclear grade in early breast cancer. Mod. Pathol..

[20] Friedrichs K., Ruiz P., Franke F., Gille I., Terpe H.-J., Imhof B.A. (1995). High expression level of α6 integrin in human breast carcinoma is correlated with reduced survival. Cancer Res..

[21] Jones J., Royall J., Critchley D., Walker R. (1997). Modulation of myoepithelial-associated α6β4 integrin in a breast cancer cell line alters invasive potential. Exp. Cell Res..

[22] Bolós V., Gasent J.M., López-Tarruella S., Grande E. (2010). The dual kinase complex FAK-Src as a promising therapeutic target in cancer. OncoTargets Ther..

[23] Cary L.A., Guan J.-L. (1999). Focal adhesion kinase in integrin-mediated signaling. Front. Biosci..

[24] Zhao X., Guan J.-L. (2011). Focal adhesion kinase and its signaling pathways in cell migration and angiogenesis. Adv. Drug Deliv. Rev..

[25] Cary L.A., Chang J.F., Guan J.-L. (1996). Stimulation of cell migration by overexpression of focal adhesion kinase and its association with Src and Fyn. J. Cell Sci..

[26] Mitra S.K., Schlaepfer D.D. (2006). Integrin-regulated FAK–Src signaling in normal and cancer cells. Curr. Opin. Cell Biol..

[27] Wu Y., He L., Zhang L., Chen J., Yi Z., Zhang J., Liu M., Pang X. (2011). Anacardic acid (6-pentadecylsalicylic acid) inhibits tumor angiogenesis by targeting Src/FAK/Rho GTPases signaling pathway. J. Pharmacol. Exp. Ther..

[28] Trimmer C., Whitaker-Menezes D., Bonuccelli G., Milliman J.N., Daumer K.M., Aplin A.E., Pestell R.G., Sotgia F., Lisanti M.P., Capozza F. (2010). CAV1 inhibits metastatic potential in melanomas through suppression of the integrin/Src/FAK signaling pathway. Cancer Res..

[29] Gupta P., Gupta N., Fofaria N.M., Ranjan A., Srivastava S.K. (2019). HER2-mediated GLI2 stabilization promotes anoikis resistance and metastasis of breast cancer cells. Cancer Lett..

[30] Gilmore A.P. (2005). Anoikis. Cell Death Differ..

[31] Simpson C.D., Anyiwe K., Schimmer A.D. (2008). Anoikis resistance and tumor metastasis. Cancer Lett..

[32] Hanahan D., Weinberg R.A. (2011). Hallmarks of cancer: The next generation. Cell.

[33] Fantozzi A., Gruber D.C., Pisarsky L., Heck C., Kunita A., Yilmaz M., Meyer-Schaller N., Cornille K., Hopfer U., Bentires-Alj M. (2014). VEGF-mediated angiogenesis links EMT-induced cancer stemness to tumor initiation. Cancer Res..

[34] Zhao Y., Adjei A.A. (2015). Targeting angiogenesis in cancer therapy: Moving beyond vascular endothelial growth factor. Oncologist.

[35] Hicklin D.J., Ellis L.M. (2005). Role of the vascular endothelial growth factor pathway in tumor growth and angiogenesis. J. Clin. Oncol..

[36] Ashton T.M., Fokas E., Kunz-Schughart L.A., Folkes L.K., Anbalagan S., Huether M., Kelly C.J., Pirovano G., Buffa F.M., Hammond E.M. (2016). The anti-malarial atovaquone increases radiosensitivity by alleviating tumour hypoxia. Nat. Commun..

[37] Xiang M., Kim H., Ho V.T., Walker S.R., Bar-Natan M., Anahtar M., Liu S., Toniolo P.A., Kroll Y., Jones N. (2016). Gene expression-based discovery of atovaquone as a STAT3 inhibitor and anticancer agent. Blood.

[38] Gao X., Liu X., Shan W., Liu Q., Wang C., Zheng J., Yao H., Tang R., Zheng J. (2018). Anti-malarial atovaquone exhibits anti-tumor effects by inducing DNA damage in hepatocellular carcinoma. Am. J. Cancer Res..

[39] Chen D., Sun X., Zhang X., Cao J. (2018). Targeting mitochondria by anthelmintic drug atovaquone sensitizes renal cell carcinoma to chemotherapy and immunotherapy. J. Biochem. Mol. Toxicol..

[40] Gupta N., Srivastava S.K. (2019). Atovaquone: An Antiprotozoal Drug Suppresses Primary and Resistant Breast Tumor Growth by Inhibiting HER2/β-Catenin Signaling. Mol. Cancer Ther..

[41] Tester A.M., Ruangpanit N., Anderson R.L., Thompson E.W. (2000). MMP-9 secretion and MMP-2 activation distinguish invasive and metastatic sublines of a mouse mammary carcinoma system showing epithelial-mesenchymal transition traits. Clin. Exp. Metastasis.

[42] Liu C.-Y., Lin H.-H., Tang M.-J., Wang Y.-K. (2015). Vimentin contributes to epithelial-mesenchymal transition cancer cell mechanics by mediating cytoskeletal organization and focal adhesion maturation. Oncotarget.

[43] Smart C.E., Morrison B.J., Saunus J.M., Vargas A.C., Keith P., Reid L., Wockner L., Amiri M.A., Sarkar D., Simpson P.T. (2013). In vitro analysis of breast cancer cell line tumourspheres and primary human breast epithelia mammospheres demonstrates inter-and intrasphere heterogeneity. PLoS ONE.

[44] Prasad S., Ramachandran S., Gupta N., Kaushik I., Srivastava S.K. (2019). Cancer cells stemness: A doorstep to targeted therapy. Biochim. Biophys. Acta Mol. Basis Dis..

[45] Wang R., Lv Q., Meng W., Tan Q., Zhang S., Mo X., Yang X. (2014). Comparison of mammosphere formation from breast cancer cell lines and primary breast tumors. J. Thorac. Dis..

[46] Reynolds D.S., Tevis K.M., Blessing W.A., Colson Y.L., Zaman M.H., Grinstaff M.W. (2017). Breast Cancer Spheroids Reveal a Differential Cancer Stem Cell Response to Chemotherapeutic Treatment. Sci. Rep..

[47] Iglesias J.M., Beloqui I., Garcia-Garcia F., Leis O., Vazquez-Martin A., Eguiara A., Cufi S., Pavon A., Menendez J.A., Dopazo J. (2013). Mammosphere formation in breast carcinoma cell lines depends upon expression of E-cadherin. PLoS ONE.

[48] Fofaria N.M., Srivastava S.K. (2014). STAT3 induces anoikis resistance, promotes cell invasion and metastatic potential in pancreatic cancer cells. Carcinogenesis.

[49] Tao K., Fang M., Alroy J., Sahagian G.G. (2008). Imagable 4T1 model for the study of late stage breast cancer. BMC Cancer.

[50] Folkman J. (2002). Role of angiogenesis in tumor growth and metastasis. Semin. Oncol..

[51] Xu H., Eirew P., Mullaly S.C., Aparicio S. (2014). The omics of triple-negative breast cancers. Clin. Chem..

[52] Bon G., Folgiero V., Di Carlo S., Sacchi A., Falcioni R. (2007). Involvement of α 6 β 4 integrin in the mechanisms that regulate breast cancer progression. Breast Cancer Res..

[53] Lu S., Simin K., Khan A., Mercurio A.M. (2008). Analysis of integrin β4 expression in human breast cancer: Association with basal-like tumors and prognostic significance. Clin. Cancer Res..

[54] van Nimwegen M.J., van de Water B. (2007). Focal adhesion kinase: A potential target in cancer therapy. Biochem. Pharmacol..

[55] Finn R. (2008). Targeting Src in breast cancer. Ann. Oncol..

[56] Deryugina E.I., Quigley J.P. (2006). Matrix metalloproteinases and tumor metastasis. Cancer Metastasis Rev..

[57] Kim C.G., Lee H., Gupta N., Ramachandran S., Kaushik I., Srivastava S., Kim S.-H., Srivastava S.K. (2018). Role of Forkhead Box Class O proteins in cancer progression and metastasis. Semin. Cancer Biol..

[58] Messica Y., Laser-Azogui A., Volberg T., Elisha Y., Lysakovskaia K., Eils R., Gladilin E., Geiger B., Beck R. (2017). The role of vimentin in regulating cell invasive migration in dense cultures of breast carcinoma cells. Nano Lett..

[59] Eccles S.A., Welch D.R. (2007). Metastasis: Recent discoveries and novel treatment strategies. Lancet.

[60] Frisch S.M., Francis H. (1994). Disruption of epithelial cell-matrix interactions induces apoptosis. J. Cell Biol..

[61] Bielecka Z.F., Maliszewska-Olejniczak K., Safir I.J., Szczylik C., Czarnecka A.M. (2017). Three-dimensional cell culture model utilization in cancer stem cell research. Biol. Rev..

[62] Gupta P., Srivastava S.K. (2014). Inhibition of HER2-integrin signaling by Cucurbitacin B leads to in vitro and in vivo breast tumor growth suppression. Oncotarget.

[63] Lal S., Kersch C., Beeson K.A., Wu Y.J., Muldoon L.L., Neuwelt E.A. (2015). Interactions between αv-integrin and HER2 and their role in the invasive phenotype of breast cancer cells in vitro and in rat brain. PLoS ONE.

[64] Gupta N., Gupta P., Srivastava S.K. (2019). Penfluridol overcomes paclitaxel resistance in metastatic breast cancer. Sci. Rep..

[65] Ranjan A., Gupta P., Srivastava S.K. (2016). Penfluridol: An antipsychotic agent suppresses metastatic tumor growth in triple-negative breast cancer by inhibiting integrin signaling axis. Cancer Res..

[66] Verma K., Gupta N., Zang T., Wangtrakluldee P., Srivastava S.K., Penning T.M., Trippier P.C. (2018). AKR1C3 Inhibitor KV-37 Exhibits Antineoplastic Effects and Potentiates Enzalutamide in Combination Therapy in Prostate Adenocarcinoma Cells. Mol. Cancer Ther..

[67] Verma K., Zang T., Gupta N., Penning T.M., Trippier P.C. (2016). Selective AKR1C3 inhibitors potentiate chemotherapeutic activity in multiple acute myeloid leukemia (AML) cell lines. ACS Med. Chem. Lett..

[68] Lombardo Y., de Giorgio A., Coombes C.R., Stebbing J., Castellano L. (2015). Mammosphere formation assay from human breast cancer tissues and cell lines. J. Vis. Exp. JoVE.

[69] Kandala P.K., Srivastava S.K. (2012). Diindolylmethane-mediated Gli1 protein suppression induces anoikis in ovarian cancer cells in vitro and blocks tumor formation ability in vivo. J. Biol. Chem..

[70] Conley F.K. (1979). Development of a metastatic brain tumor model in mice. Cancer Res..

[71] Gupta P., Adkins C., Lockman P., Srivastava S.K. (2013). Metastasis of breast tumor cells to brain is suppressed by phenethyl isothiocyanate in a novel in vivo metastasis model. PLoS ONE.

[72] Boreddy S.R., Sahu R.P., Srivastava S.K. (2011). Benzyl isothiocyanate suppresses pancreatic tumor angiogenesis and invasion by inhibiting HIF-alpha/VEGF/Rho-GTPases: Pivotal role of STAT-3. PLoS ONE.

