# Supplementary material for: Atovaquone Suppresses the Growth of Metastatic Triple-Negative Breast Tumors in Lungs and Brain by Inhibiting Integrin/FAK Signaling Axis
Source: Pharmaceuticals (Basel). 2021 May 28;14(6):521. doi: 10.3390/ph14060521 (PMC8229709; doi:10.3390/ph14060521)
Supplement: Supplementary file 1 [file pharmaceuticals-14-00521-s001.zip › pharmaceuticals-1126162-supplementary.pptx]

## Slide 1
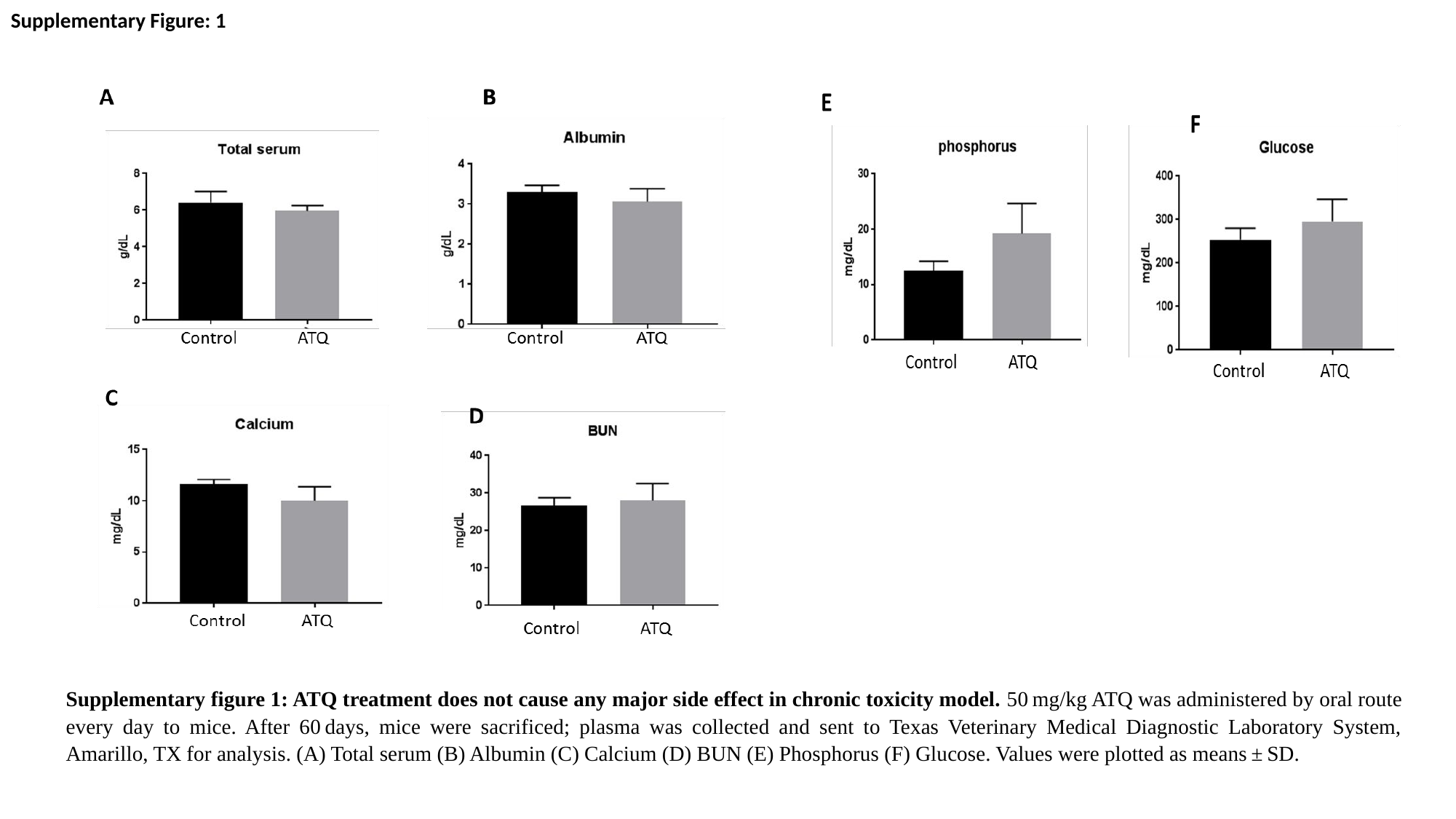

Supplementary Figure: 1
Supplementary figure 1: ATQ treatment does not cause any major side effect in chronic toxicity model. 50 mg/kg ATQ was administered by oral route every day to mice. After 60 days, mice were sacrificed; plasma was collected and sent to Texas Veterinary Medical Diagnostic Laboratory System, Amarillo, TX for analysis. (A) Total serum (B) Albumin (C) Calcium (D) BUN (E) Phosphorus (F) Glucose. Values were plotted as means ± SD.

## Slide 2
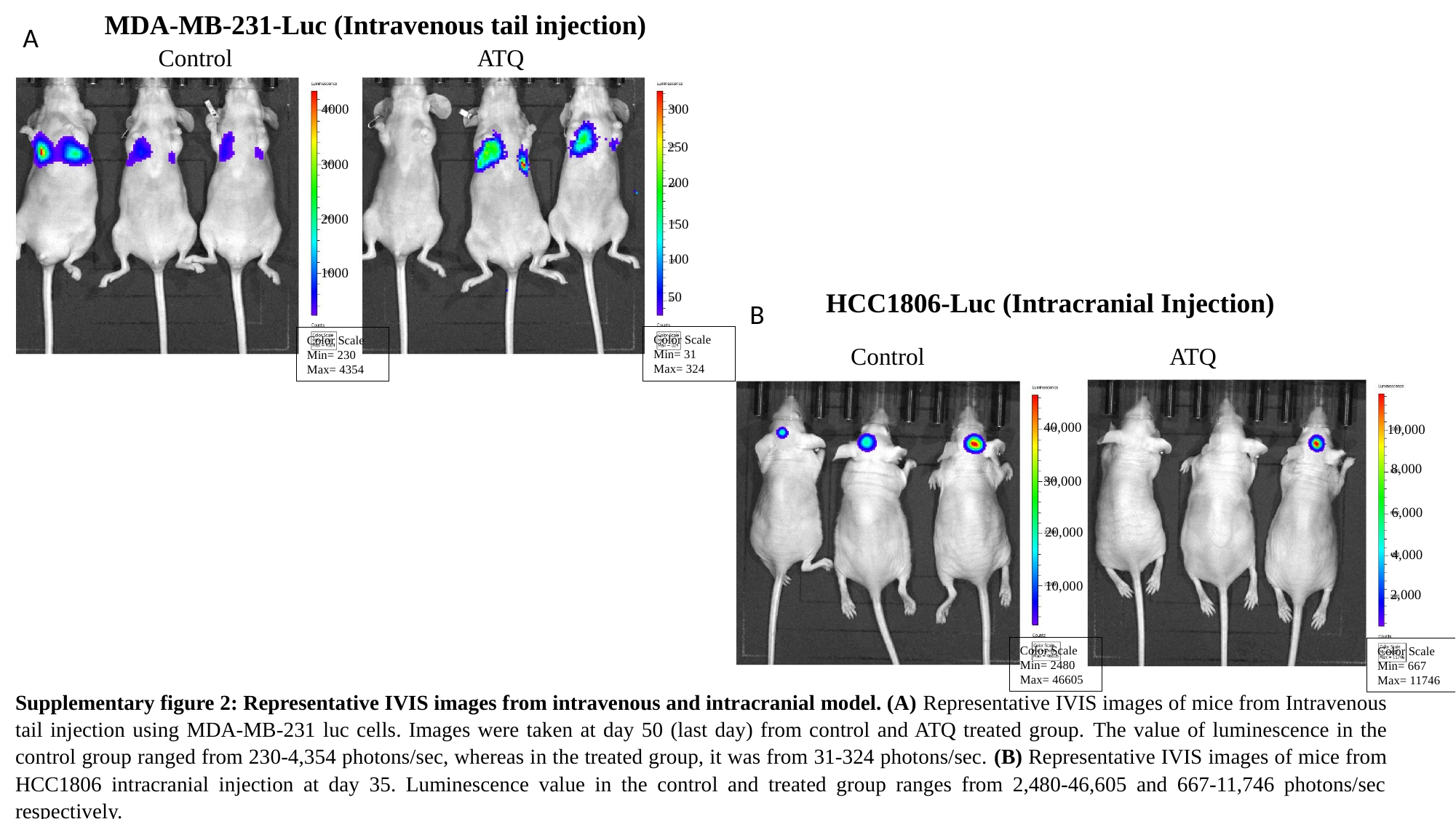

MDA-MB-231-Luc (Intravenous tail injection)
A
Control ATQ
4000
300
250
3000
200
2000
150
100
1000
50
Color Scale
Min= 31
Max= 324
Color Scale
Min= 230
Max= 4354
HCC1806-Luc (Intracranial Injection)
B
Control ATQ
40,000
30,000
20,000
10,000
10,000
8,000
6,000
4,000
2,000
Color Scale
Min= 2480
Max= 46605
Color Scale
Min= 667
Max= 11746
Supplementary figure 2: Representative IVIS images from intravenous and intracranial model. (A) Representative IVIS images of mice from Intravenous tail injection using MDA-MB-231 luc cells. Images were taken at day 50 (last day) from control and ATQ treated group. The value of luminescence in the control group ranged from 230-4,354 photons/sec, whereas in the treated group, it was from 31-324 photons/sec. (B) Representative IVIS images of mice from HCC1806 intracranial injection at day 35. Luminescence value in the control and treated group ranges from 2,480-46,605 and 667-11,746 photons/sec respectively.
